# Another District Medical Society Organized

**Published:** 1900-10

**Authors:** 


### Another District Medical Society Organized.

GRAHAM, TEXAS, September 10, 1900.

*Texas Medical Journal, Austin, Texas:*

The Young County Medical Society has reorganized and formed a district society, to be known as the Young County and District Medical Society. It is to include Young and adjoining counties.

The first district meeting was held on the second Wednesday in July The following officers were elected: Dr. W. M. Terrell, president; Dr. L. A. Loggins, vice-president; Dr. G. F. LeGrand, treasurer; and Dr. L. LeGrand, secretary.

All reputable physicians who are graduates of regular recognized medical schools are eligible to membership.

The next quarterly meeting occurs at Graham on the second Wednesday in October, and the program is one calculated to be of unusual interest to those of this locality. It is expected to be well attended.

